# Navigating Diabetes: Enhancing Self-Management through Education among Diabetic People at the Early Stages of the Disease—A Systematic Review

**DOI:** 10.3390/ijerph21050522

**Published:** 2024-04-23

**Authors:** Emirjona Kiçaj, Aurela Saliaj, Rudina Çerçizaj, Vasilika Prifti, Sonila Qirko, Liliana Rogozea

**Affiliations:** 1 Faculty of Medicine, Transylvania University, 500019 Brasov, Romania; rudina.cercizaj@unitbv.ro (R.Ç.); vasilika.prifti@unitbv.ro (V.P.); sonila.qirko@unitbv.ro (S.Q.); r_liliana@unitbv.ro (L.R.); 2 Faculty of Health, University “Ismail Qemali” Vlore, 9401 Vlore, Albania; aurela.dai@univlora.edu.al

**Keywords:** diabetic patients, T2DM, health education, diabetes self-management, early-stage disease

## Abstract

Diabetes self-management education helps to improve health outcomes and qualities of life for diabetic patients. This systematic review examines the effectiveness of several types of diabetes self-management education for patients at the early stages of type 2 diabetes mellitus (T2DM). A review of studies that have researched the use and impacts of health education on diabetic patients with T2DM was conducted using the electronic databases PubMed, Elsevier, JSTORE, Walters Kluwer, and the Cochrane Library between January 2017 and November 2022. We found 789 studies, and after selecting the PRISMA flowchart, we selected 19 studies, including those of 2512 adult patients diagnosed with T2DM. Biomedical results presented the pooled effect of a glycated hemoglobin (HbA1c) of −0.64% and a fasting blood glucose (FBG) of −0.32. Emotional and social results and behavioral effects were evaluated in 10 and nine studies, respectively. The education and support of diabetic patients at the early stages of the disease impact various aspects, including the biomedical profile, lifestyle, emotional and social well-being, and anthropometric parameters. Among the factors that have been identified to enhance the effectiveness of educational interventions are the following: conducting individualized sessions (or at least in small groups of patients), extending the duration of interventions by at least 12 months, adopting a combined approach that includes both face-to-face and online components, and ensuring the involvement of a multidisciplinary healthcare team.

## 1. Introduction

Diabetes mellitus is mentioned as being among the major health problems in the world in terms of its widespread occurrence, the impact it has on socioeconomic development, and the severe influence it has on the quality of life of patients [1]. According to the International Diabetes Federation (IDF), in 2019, the number of diabetic adults aged 20–79 years was approximately 463 million, a number predicted to increase to 700 million individuals worldwide by 2045, with 90% of the cases being diabetes mellitus type 2 (T2DM) [2,3,4]. The IDF reported that although the incidence of T2DM is decreasing or stable in developed countries [2], a rapid increase in T2DM has been observed in developing countries [5]. An increasing number of effective treatments should be sought for diabetes, and the goal of the United Nations (UN) is to reduce premature deaths from non-communicable diseases, including diabetes [6]. Glucose control is the cornerstone of T2DM treatment, but crucial aspects of treatment are also the implementation of programs that include lifestyle modification, the careful use of oral anti-hyperglycemic medications, and the initiation of insulin when necessary [7,8,9,10]. Patients with type 2 diabetes should receive professional guidance to improve their self-care behaviors, leading to improved glucose control [11]. The four best times to provide this guidance are at the time of the diagnosis, annually or when objectives are not met, upon the emergence of factors influencing complications, and during significant life care transitions [12].

The utilization of diabetes self-management education contributes to enhanced health outcomes, the quality of care, and the overall quality of life for diabetic patients, ultimately leading to reduced expenses and bringing about positive changes in lifestyle and self-care management [12,13,14]. Implementing lifestyle interventions in newly diagnosed diabetic patients with T2DM leads to improvements in cardiometabolic parameters, offering long-term health and well-being benefits [15]. According to the American Diabetes Association (ADA), individuals diagnosed with diabetes should receive comprehensive information and guidance at the time of the diagnosis, with ongoing education and support thereafter [12]. The moment of the diagnosis serves as a critical juncture when patients actively seek information about their new health situation and must adapt to new health behaviors [16]. The support required to implement and sustain coping skills and behaviors needs to be on an ongoing basis, helped by social groups, and provided by healthcare professionals [17,18].

A previous systematic review has examined the overall impact of diabetes self-management among newly diagnosed patients with type 2 diabetes mellitus (T2DM). However, it is essential to conduct a critical appraisal to discern various educational approaches and their effects on patients with T2DM in the early stages of the disease [19].

Our systematic review aims to address this gap by evaluating the effectiveness of several types of diabetes self-management education specifically tailored to this population. We aim to identify key elements that can enhance educational interventions. By synthesizing the latest evidence, our study seeks to provide crucial insights that can assist healthcare professionals and policymakers in improving diabetes education programs for individuals at the early stages of the disease. Ultimately, our goal is to enhance health outcomes and quality of life.

## 2. Materials and Methods

### 2.1. Study Design

This is a systematic review study.

### 2.2. Search Methods

A literature search was conducted using the electronic databases of PubMed, Scopus, Elsevier, JSTORE, Walter Kluwer, and the Cochrane Library. This search was conducted for four months from August to November 2022, using the elements of the PICO model (P—population/patients; I—intervention; C—comparator/control; and O—outcomes). The search included the use of the following keywords:

– Population-related terms: “diabetes mellitus type 2”, “diabetes mellitus II”, “type 2 diabetic patients”, “patients with T2DM”, “patients diagnosed within the last 0–5 years”;
– Intervention-related terms: “educational intervention”, “diabetes self-management education program evaluation”, “diabetes self-management program effectiveness”, “diabetes self-care education”, and “lifestyle intervention”;
– Comparator/control-related terms: “assessing changes in the intervention group (IG) and control group (CG)” and “evaluating changes in IG (intervention group) and CG (control group)”;
– Outcome-related terms: “assessment”, “evaluation”, “examination”, “measurement”.

This study was conducted using the PRISMA guidelines for reporting systematic reviews [20].

We conducted a thorough electronic search, carefully applying filters to ensure the inclusion of articles that genuinely cater to the well-being of adults with T2DM. The focus of these articles encompasses various aspects, including educational interventions, such as multi-intervention programs, nutritional literacy or health literacy, and face-to-face or online intervention. Furthermore, we sought studies that not only assessed these educational interventions but also provided insights into measured outcomes, spanning biomedical, behavioral, emotional, and social improvements. This approach reflects our commitment to understand and address the holistic needs of individuals with T2DM.

We limited the inclusion criteria based on the participants’ ages (participants had to be older than 18 years), articles published in English between 2017 and 2022, and availability of full-text publications in peer-reviewed journals. This selection aimed to ensure a thoughtful and focused approach, considering the accessibility and language proficiency of the content while also respecting the ethical considerations associated with participants’ ages. The inclusion and exclusion criteria are presented in Table 1.

We manually searched the reference lists of pertinent publications to identify relevant articles according to our inclusion and exclusion criteria.

Following the removal of duplicate results, two researchers (E.K. and A.S.) individually reviewed the titles and abstracts to identify the most suitable articles. Collaboratively, they referred to the screening process and addressed any discrepancies and then proceeded to independently read the abstracts and select the most relevant ones.

Throughout this screening phase, any ambiguities were resolved through group discussion among the researchers, ensuring alignment with the eligibility criteria established for including or excluding articles.

**Figure 1:**
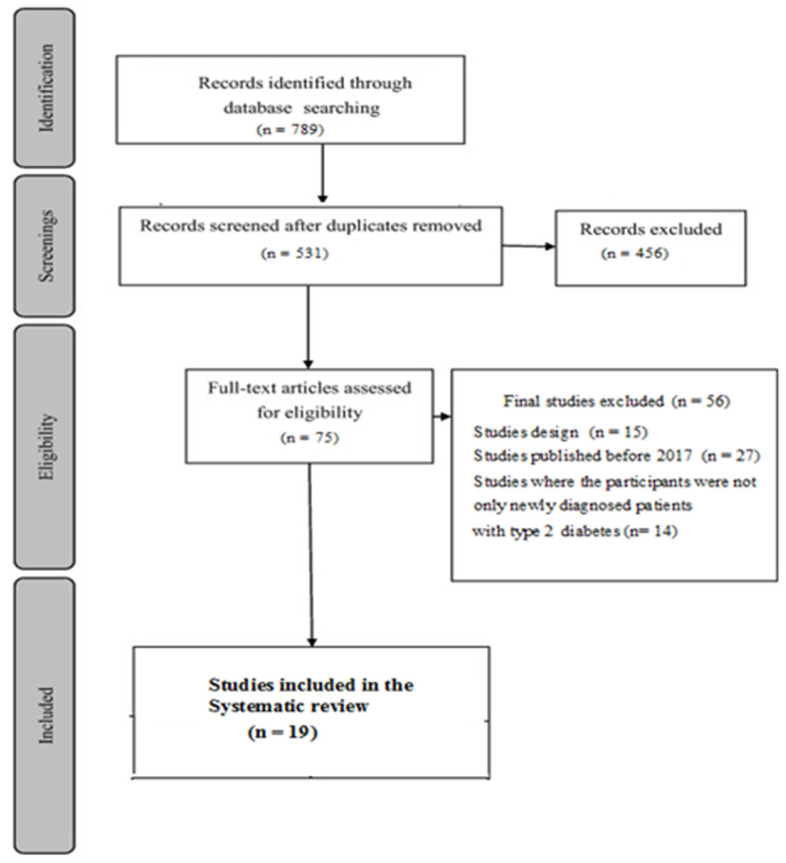
The PRISMA flowchart of included and excluded studies.

Figure 1 displays the PRISMA flow diagram, depicting the systematic review process for searching for and selecting studies for inclusion.

### 2.3. Data Extraction

All the data were extracted by two researchers (E.K. and A.S.). The data included the details of the publications (title, authors, journal, year of publication, and country of origin), methods (the aim of the study, design, and duration of the intervention), participants (number of patients in the intervention group and in the control group, age, and sex), interventions (type of intervention, settings, descriptions of the intervention and the standard care, the duration of the intervention, timing, delivery, method of follow-up, providers), and outcomes (clinical parameters and psychological and behavioral outcomes).

### 2.4. Synthesis of the Results 

A summary of the information from the studies included in this analysis was carried out according to the place of the study, population, duration of education, and details about the educational interventions, such as the method of delivery, the people who carried out the educational interventions, the theoretical materials offered, the frequency and duration of the educational sessions, and a summary of the outcomes of these interventions among diabetic patients. The studies included in this review had different interventions and durations. For this, we have made a narrative summary, presenting the clinical results (HbA1, FBG, postprandial blood glucose—PBG, anthropometric parameters, and blood pressure) using means ± standard deviation.

Risk of Bias: Eleven (57.89%) of the included studies had poor quality. This assessment was carried out following the revised Cochrane risk-of-bias tool [21]. Most of the studies were conducted without blinding the participants and personnel because of the nature of educational interventions (Table 2).

## 3. Results

Nineteen studies met the inclusion criteria established in advance for this review study.

### 3.1. Overview of the Studies

The evaluated participants’ characteristics and interventions are presented in Table 3 and Table 4, respectively. These studies were conducted in different countries, such as China [22,23,24,25,26,27], India [28,29,30,31], and the Netherlands [32,33] and one study each in Spain [34], Mexico [35] Italy [36], USA [37], the United Kingdom [38], Germany [39], and Lebanon [40].

This study encompassed 2512 newly diagnosed diabetic patients with a sample size ranging from 17 to 358 patients. The mean age range of the participants was from 25 to 74 years, with 75% of the studies involving a population of over 50 years old. The mean duration of the diabetes ranged from 3 months to 5 years (Table 3).

While analyzing the studies, various interventions were identified, each characterized by distinct features, durations, and assessment methods. The purpose of the included studies was to evaluate the impacts of various educational interventions within a period from 1.4 to 30 months. We included 19 studies, of which 16 used methods for comparing interventions with a control group, while three of them did not use any control group. Among the 19 studies, nine were randomized controlled trials (RCTs) [22,26,28,29,31,32,33,38], one was an experimental study [23], four were intervention studies [24,36,37,39], two were program evaluation studies [35,40], two were prospective studies [27,34], and one was a non-randomized controlled study [25].

The interventions assessed in these studies include a multi-intervention program (73.68%) [22,23,24,25,26,27,28,29,32,33,34,35,37,38], physical activity intervention (10.5%) [30,31], nutritional intervention (10.5%) [39,40], and a single study that evaluated psychological interventions [36]. Educational interventions included groups of participants in 47.36% of the studies, individual interventions in 47.36%, and a combination of the two in only one study (Table 4).

In terms of intervention methods, 26.3% of the studies utilized telephone applications, and one study used text messages directed to the participants. Face-to-face interventions were implemented in most studies (68.4%). Educational interventions for newly diagnosed diabetic patients with T2DM were conducted by different healthcare professionals (physicians, nurses, dieticians, pharmacists, and diabetes educators). Twenty-one percent of the studies [22,25,34,35] included educational interventions delivered by a team of more than one member, including a nurse. Meanwhile, nurses delivered educational interventions in 26.3% of the studies [23,24,26] (Table 4).

The evaluation included five programs that were evaluated as the AADE 7 Self-Care Behaviors program [22] PAET-Debut DM2 program [34] Omaha System-based integrated nursing management model [25], CAIPadi model [35], and nurse-led integrative-medicine-based structured education program–multi intervention program [26] (Table 4).

Biomedical outcomes were reported in 16 studies; psychosocial outcomes, in 12 studies; behavioral outcomes, in 10 studies. Six studies included elements in all the outcome categories [26,28,32,34,35,37].

### 3.2. Impacts of Educational Interventions on Biomedical Results

#### 3.2.1. The Impacts of Educational Interventions on HbA1c Levels (Table 5)

The effects of educational interventions on HbA1c levels were evaluated in 12 studies (60%) [22,23,26,27,28,30,32,34,35,36,37,40]. A comparison of the results between the intervention and control groups is presented in nine studies, where we evaluated the absolute effect. Improvement in HbA1c values was significant in the intervention group in 10 (83.3% of the) studies and in the control group in three studies. Six studies showed significant differences in the effects on HbA1c values between the two study groups (IG and CG). Notably, in a study conducted in the Netherlands, there was no evident change in HbA1c levels after the intervention; in fact, there was a very slight increase (0.1%) in HBa1c values [32]. The differences in the intervention and control groups were 1.18% and 0.277%, respectively. The absolute effect of the educational intervention on HbA1c was −0.64.

**Table 5:** The effects of educational interventions on HbA1c, FBG, and PGB.

Studies’ General Information	Pre-Intervention		Post-Intervention		Change		Absolute Effect
	IG	CG	IG	CG	IG	CG	
Effect of educational intervention on HbA1c							
[22]	8.44 ± 2.28	8.95 ± 2.34	6.92 ± 1.27 *	7.82 ± 12.98 *^#^	−1.52 ^$^	−1.13	−0.39
[23]	7.20 (6.40, 9.10)	7.90 (6.80, 10.30)	6.20 (5.80, 6.60) *	6.70 (6.40, 7.30) *	−1 **	−1.2	+0.2
[26]	6.66 ± 1.09	6.86 ± 1.34	5.85 ± 0.37 *	6.97 ± 1.18	−0.81 **	+0.11	−0.70
[27]	9.82 ± 2.47	9.05 ± 2.32	6.76 ± 0.50 *	7.25 ± 0.98 *	−3.03 **	−1.8	−1.23
[28]	9.5 ± 2.1	9.5 ± 2.0	7.4 ± 1.5 *	9.5 ± 2.1	−2.1 **	0	−2.1
[30]	5.95 ± 0.47	5.95 ± 0.47	5.14 ± 0.36 *	5.85 ± 0.37 *	−0.81 **	−0.1	−0.71
[32]	6.5 (6.2–7.0)	6.6 (6.3–7.1)	6.6 (6.3–7.1)	6.7 (6.3–7.1)	+0.1 *^#^	+0.1	0
[34]	7.2 (6.6–9.2)	6.7 (6.4–7.5)	6.2 (5.8–6.7) *	6.4 (5.8–6.8)	−1 ^$^	−0.3	−0.7
[35]	7.77 ± 2.22	No control group	7.16 ± 1.62	No control group	−0.61 *^#^	−	−0.61
[36]	7.85 ± 1.19	7.32 ± 1.23	6.66 ± 0.84 *	6.95 ± 1.31	−1.19 **	−0.37	−0.82
[37]	8.0 (1.6)	-	6.2 (1.1) *	-	−1.8 *^#^	−	−1.6 ± 0.5
[40]	9.1 ± 2.3	-	7.4 ± 1.3 *	-	−1.7 ± 2.5 *	−	−1.7 ± 1
Mean ± SD					−1.18 ± 0.21	−0.277 ± 0.13	−0.64 ± 0.08
Effect of educational intervention on FBG							
[23]	8.00	8.00	6.78 *	7.70 *	−1.22 ^$^	−0.3	−0.92
[24]	8.43 ± 1.25	8.51 ± 1.17	7.03 ± 1.01 *	7.68 ± 1.12	−1.4 **	−0.83	−0.57
[25]	9.964 ± 2.707	10.490 ± 2.781	7.792 ± 0.925 *	9.042 ± 1.561	−2.172 **	−1.448	−0.72
[28]	10.9 ± 3.6	11.5 ± 3.9	7.5 ± 2.3 *	8.4 ± 2.8 *	−3.4 **	−3.1	−0.3
[32]	7.4	7.3	7.9	7.5	+0.5 *^#^	+0.2 *^#^	+0.3
[38]	5.75 ± 1.01	6.55 ± 1.76	5.66 ± 1.20	6.73 ± 2.66	−0.09 *^#^	+0.18	0.27
Mean ± SD					−1.656 ± 2.11	−2.839 ± 2.31	−0.32 ± 1.16
Effect of educational intervention on PBG							
[23]	13.29	12.67	7.90 *	10.58 *	−5.39 ^$^	−2.09	−3.3
[24]	11.21 ± 1.65	11.34 ± 1.73	9.52 ± 1.05 *	10.43 ± 1.24	−1.69 **	−0.91	−0.78
[25]	14.612 ± 4.685	14.692 ± 4.400	9.980 ± 1.446 *	12.275 ± 2.120	−4.632 **	−2.417	−2.215
[28]	17.1 ± 4.6	17.2 ± 4.9	12.5 ± 3.3 *	12.7 ± 3.6 *	−4.6 *^#^	−4.5	−0.1
Mean ± SD					−4.078 ± 2.35	−2.479 ± 2.41	−1.598 ± 0.23

Notes: * Significant difference between pre- and post-intervention in the same group. *^#^ Non-significant difference between pre- and post-intervention in the same group. ** Significantly different effects between IG and CG. ^$^ No statistical comparison or no data about significance. Studies without control group are not included in the calculation of absolute effects on HbA1c, FBG, and PBG levels.

Educational interventions demonstrate visible effects in reducing HbA1c levels, particularly in cases with fewer patients attending educational sessions and interventions lasting 12 months [34,36,40]. Additionally, a combination of face-to-face and online educational methods [22,28,40] has proven to be effective.

Concerning the personnel involved in the educational interventions, cases with only one type of professional yielded more satisfactory results (The absolute effect on the level of HbA1c was −0.87 ± 0.67). When interventions were conducted by nurses, the absolute effect was −0.50 ± 0.15. Conversely, when interventions were carried out by a team, the absolute effect was −0.56 ± 0.78.

Individual interventions emerged as the most efficient in 55% of the studies assessing HbA1c levels. These individual interventions demonstrated a notable improvement in the HbA1 level by 1.12% [22,27,28,30,35,40], while group-based education resulted in a less pronounced effect of 0.50%.

#### 3.2.2. The impacts of Educational Interventions on FBG and PBG Values

Seven studies (35%) assessed the impacts of educational interventions on FBG levels [23,24,25,28,32,38]. In one study, very slight increases in FBG values were observed in both the intervention and control groups [32]. Significant changes in FBG values within the intervention group were reported in four studies [23,24,25,28], whereas this difference was significant in the control group in one of these studies [28]. Notably, significantly different effects between intervention and control groups were observed in three studies [24,25,28].

Four studies (20%) investigated the effects of educational interventions on PBG levels [23,24,25,28]. All these studies demonstrated a significant difference between pre- and post-intervention levels in the intervention group, with significant differences observed between the intervention and control groups in only two studies [24,25]. Collectively, the mean change (improvement) in FBG levels after the intervention was 0.32% and in PBG levels was 1.59%. The absolute effect of the educational intervention on FBG was −0.32 ± 1.16 and on PBG was −1.598 ± 0.23 (Table 5).

#### 3.2.3. The Impacts of Educational Interventions on Lipid Profiles

Five studies examined the impacts of educational interventions on lipid profiles (total cholesterol (T-Chol), low-density lipoprotein cholesterol (LDL-C), high-density lipoprotein cholesterol (HDLC), and triglycerides (TGs)) [23,32,34,39,40]. These studies used different units of measurement to assess lipid levels, making it impossible to statistically evaluate the mean ± SD.

Significant differences in T-Chol between pre- and post-intervention in the intervention group were observed in two studies [34,39] and for LDL-C in two studies [32,39]. Changes in HDL-C and triglyceride levels were not statistically significant.

#### 3.2.4. The impacts of Educational Interventions on Anthropometric Parameters

The impacts of educational interventions on the bodyweights of diabetic patients with T2DM were assessed in five studies [22,31,32,38,39] and on body mass indices (BMIs) in 11 studies [22,23,27,31,32,34,35,37,38,39,40].

Four studies revealed a decrease in bodyweight, but only one study reported a significant change in body weight [22]. The absolute effect of educational interventions, among studies with two groups (intervention and control groups), on weight was 2.94%.

Of the 11 studies evaluating the effects of educational interventions on the BMIs of diabetic patients, five studies did not compare the results with a control group [34,35,37,39,40]. BMI decreased in both groups without a significant intervention effect, except for one study in which the effect size was insignificant or small [38]. The absolute effect of educational interventions among studies with two groups (intervention group and control group) on BMI was 0.39% (Table 6).

#### 3.2.5. The Impacts of Educational Interventions on Blood Pressures

Eight studies [22,23,31,32,34,35,38,40] evaluated the impacts of educational interventions on arterial pressures among newly diagnosed patients with T2DM, and six compared arterial pressure values between the two study groups (intervention and control groups) [22,23,31,32,38]. Only one study reported a statistically significant difference in systolic blood pressure (SBP) and diastolic blood pressure (DBP) [40].

The mean changes in the SBP and DBP of the intervention group were −2.3 ± 6.5 mmHg and −0.87 ± 4.5 mmHg, respectively. The absolute effect of educational interventions on the SBP was −0.34 ± 7.9 mmHg and on the DBP was −0.36 ± 5.5 mmHg (Table 7).

### 3.3. The impacts of Educational Interventions on Emotional and Social Results

To assess the emotional and social impacts of educational interventions among newly diagnosed diabetics, we collected information on improvements in knowledge, illness perception, anxiety and depression, diabetes distress, empowerment, diabetes self-efficacy, diabetes self-management, and quality of life.

Five studies evaluated the impacts of educational interventions on the knowledge of patients with diabetes, using different assessment tools [22,25,29]. All five studies reported improvements in knowledge at the end of the interventions, with a noticeable significant difference in knowledge between the intervention group (IG) and the control group (CG) stated in three particular studies [22,25,29].

Among these studies, only one assessed illness perception using the “Illness Perceptions Questionnaire” (IPQ-R). Immediately after the education program, the intervention group showed a significantly higher belief in having diabetes than did the control group [33].

Depression and anxiety were evaluated in three studies; anxiety, in two studies [23,35]; depression, in three studies [23,35,37]. All these studies revealed that educational interventions positively reduced anxiety, depression, and other symptoms in patients with T2DM.

Two studies showed significant improvements in diabetes distress as a result of educational interventions [35,37], whereas another study did not show any improvement in this aspect [33].

Diabetic patient empowerment was assessed in three studies using different assessment methods, such as the Diabetes Empowerment Scale-Short Form [DESSF] [35] and the Diabetes Empowerment Scale (DES) [33,37]. All three of these studies reported significant improvements in the intervention groups.

Quality of life was evaluated in six studies [24,25,28,32,34,35], with two of them lacking a data comparison between the intervention and control groups. The instruments used to evaluate interventions in the quality of life were SF-36, EuroQol-5d, Diabetes-Specific Quality-of-Life scale (DSQL), Diabetes Quality-of-Life Measure (DQoL), and Diabetes-Dependent Quality-of-Life (ADDQoL). In four studies [24,25,34,35], significant improvements were observed in the quality of life of patients with T2DM, whereas in the other two studies, patients presented negative effects that did not change over time [28,32].

### 3.4. The Impacts of Educational Interventions on Behavioral Results

The studies included in this analysis employed diverse assessment methods.

Three studies assessed the changes in tobacco use. In one study, there were moderate but significant reductions in smoking and alcohol consumption [34]. In two other studies, a moderate reduction in tobacco use was observed, although the difference was not statistically significant [26,33].

Eight studies reported physical activity, assessed in six studies through standardized questionnaires [26,28,31,32,33,35], the use of a mobile application [38], or through subjective measures [40]. Hernandez et al., reported an improvement in physical activity [35], while in other studies, these changes were not significant between the intervention and control groups.

Changes in dietary behavior were reported in five studies. In four of them, the impact of educational interventions was evaluated through the use of validated methods [26,32,33,34] and the self-reporting of patients regarding the use of high- and low-carbohydrate foods [37]. Initially, changes in two of these studies were not significant [32,34]. Participants in the intervention group exhibited significantly better self-management behaviors related to the intakes of fruits and vegetables at both the immediate post-intervention and 12th-week follow-ups [32]. Meanwhile, in another study, although immediately after the program was used [33], the results showed an increase in the consumption of fruits and vegetables, these effects were no longer present six months after the interventions. Oser et al. reported satisfactory results in terms of reducing the use of high-carbohydrate foods, even three months after the interventions [37].

The self-care activity has been evaluated in five studies, all of which presented a positive impact of educational interventions, thereby increasing patients’ awareness [26,32,33,34,35].

## 4. Discussion

The results of this study reveal the effectiveness of educational interventions implemented for newly diagnosed patients with T2DM to promote the importance of healthcare education since the beginning of the diagnosis.

In this systematic review, we uncovered compelling evidence supporting the effectiveness of diabetes self-management education during the early stages of type 2 diabetes mellitus (T2DM). Our analysis delineated the variances among several types of educational approaches: individual versus team training, personalized versus group counseling, short-term versus ongoing support, and online versus face-to-face interaction. We observed how these factors contributed to enhanced glucose control and improved health outcomes among this population.

Early educational interventions possess the potential to empower patients to embrace healthy behaviors and self-care practices, thereby mitigating the risk of complications associated with T2DM.

Based on the overall analysis of the impacts of various educational interventions for diabetic patients regarding their clinical or other parameters, significant results were found.

According to the American Diabetes Association (ADA), educational interventions can cause a reduction by 1% in HbA1c levels among diabetic patients [17].

Our results showed that there was a significant improvement in HbA1c levels in the intervention group in most of the included studies, with an absolute effect size of −0.64%, a lower result compared to another study conducted in 2020 [41] but slightly higher compared to two other reviews, one in 2020, with an absolute effect of 0.21% [32,33,34,35]. The factors, mentioned in these studies, that improve the control of glycemia are the implementation periods of educational interventions; use of various techniques, such as the combination of face-to-face with online methods; use of different intervention strategies; and use of means to help to achieve expected results, such as online applications or providing supportive materials for patients to improve their knowledge of diabetes and glycemic control.

Regarding clinical parameters, four studies assessed changes in an FBG of 0.32 and changes in a PBG of 1.59. However, the results for lipid profile changes were inconclusive because of variations in the measurement units.

Anthropometric parameters, specifically the BMI, showed a difference of 0.87% in 11 studies and a 0.39% difference in the intervention group. The changes in the arterial pressure were not statistically significant.

Nine studies evaluated changes in HbA1c compared to a control group, revealing that better changes were achieved when the interventions lasted for more than one year [34,36], were conducted face-to-face, and were implemented by one healthcare professional. When we compared the absolute effect on HbA1c levels for different approaches of educational interventions, the model of face-to-face combined with online interventions, such as a text message or a mobile application, offered better results in glycemic control.

Support from healthcare teams is crucial for diabetes management [42]. The most interesting result in our review was that better results were achieved when the intervention was conducted by one healthcare professional compared with the interventions offered by a team of healthcare professionals. This result is similar to that in another review that concluded that the healthcare education offered by pharmacists improved the clinical results of newly diagnosed diabetic patients [43]. The factors that may have impacted the improvement could have been the consistent approach, expertise of the professionals, clear and easy communication, and instructions only with one individual, and personalized attention.

Our systematic review presented the need to implement educational interventions individually, as they improved the control of the hyperglycemia. Additionally, Odgers-Jewelle et al. suggested that group-based diabetes self-management education is related to improved clinical and psychosocial results in people with type 2 diabetes [44]. However other studies have concluded that individual and group interventions show positive clinical results [45].

Our study found that there were more significant improvements in HbA1c levels in studies in which educational interventions were provided by one healthcare specialist. This result is consistent with a study that showed that individual-based education can achieve greater glycemic improvement than team-based education [41].

Educational interventions demonstrated modest effects on the BMIs and bodyweights of diabetic patients, exhibiting noticeable reductions in both parameters, especially in studies associated with individual interventions. These parameters, especially the body mass index (BMI), showed a difference of 0.87% in 11 studies and a 0.39% difference in the intervention group.

The duration of the education is important to obtain better results in glycemic control in diabetes management. According to the ADA, from 6 to 12 months is the best time duration for educational sessions among diabetes patients [46]. Our study found that we had significant improvement in HbA1c levels in those cases where patients’ education was followed for 12 months.

Regarding emotional and social findings, six studies assessed knowledge levels, showing significant improvements in the intervention groups. Similar results were reported in a previous study [41]. Furthermore, 50% of the studies focused on emotional and social aspects, with noticeable changes in anxiety, illness perception, empowerment, depression, diabetes distress, diabetes self-efficacy, and quality of life.

Patients recently diagnosed with T2DM have a better tendency to engage in positive behavioral changes [44]. In our systematic review, behavioral results indicated positive changes, including lifestyle changes, such as reductions in smoking and alcohol use. Physical activity was found in eight studies, while adherence to healthy diets was evaluated in five studies. Significant changes were reported immediately after the intervention, but these effects were not sustained over time. A result similar to ours was presented by Tanaka et al. [15].

This systematic review provides valuable information regarding the effectiveness of educational interventions among diagnosed diabetes patients at the early stages of the disease, treating various aspects, contents of education, and factors that impact the achievement of better results. This study has both strengths and weaknesses. This study followed the Preferred Reporting Items for Systematic Reviews guidelines to collect and evaluate the collected studies, but we only used some electronic databases. A meta-analysis could not be performed because of the heterogenicity of the data and methods that were used. Populations included in the study were from different parts of the world and countries with significant changes in their economic development, which could be a reason for the heterogeneous results.

## 5. Conclusions

In conclusion, our research highlights the profound influence that educational interventions by healthcare professionals can have on the management of type 2 diabetes mellitus (T2DM) in the early stages of diabetes. These interventions impact various aspects, including biomedical profiles, lifestyles, emotional and social well-beings, as well as anthropometric parameters.

Among the factors that have been identified to enhance the effectiveness of educational interventions are the following: conducting individualized sessions (or at least in small groups of patients), extending the duration of interventions to at least 12 months, adopting a combined approach that includes both face-to-face and online components, and ensuring the involvement of a multidisciplinary healthcare team.

Researchers should assess the sustainability of educational interventions, thus evaluating the long-term effects and preservation of knowledge in the long run, behavioral changes, and improvement in clinical results longer than the period of the healthcare education.

## Figures and Tables

**Table 1:** Inclusion and exclusion criteria.

	Inclusion Criteria	Exclusion Criteria
Publication Date	January 2017–November 2022	<2017
Study Design	RCTs ^1^ and non-randomized controlled studies, intervention studies, and cohort studies	Nonintervention studies ^2^
Population	Adults ≥ 18 years old, diabetic patients Patients at the early stages of the disease ^3^ ≤5 years from diagnosis with an oral hypoglycemic agent and lifestyle intervention	Children, not newly diagnosed T2DM patients ≥5 years from diagnosis with type 2 diabetes
Intervention	Different interventions are used for health education. Face-to-face intervention -Online intervention No changes in medication treatment during the study	Drug-based intervention with changes in medication treatment
Control Group	Standard care, routine care if it is present	
Effectiveness Assessment	Biomedical results Behavioral results Emotional and social results	

^1^ RCTs—randomized controlled studies; ^2^ nonintervention studies—qualitative studies, case control studies, review studies, and observational studies. ^3^ Early stages of disease include recent diagnoses (0–1 year after diagnosis) and short-term diagnoses (1–5 years after diagnosis).

**Table 2:** Assessment of bias risk in the included studies.

Studies’ General Information	Random Sequence Generation	Allocation Concealment	Blinding of Participants and Personnel	Blinding of Outcome Assessment	Incomplete Outcome Data	Selective Reporting	Other Biases	Study Quality
[22] (2019) China	+	+	−	?	+	?	+	Fair
[23] (2018) China	?	?	−	?	+	+	+	Poor
[24] (2018) China	+	?	?	?	+	?	+	Poor
[25] (2019) China	−	?	?	+	+	+	−	Poor
[26] (2022) China	+	+	+	+	+	+	?	Fair
[27] (2018) China	−	?	?	+	+	+	?	Poor
[28] (2019) India	+	+	+	+	+	+	+	Fair
[29] (2018) India	?	+	−	?	+	−	+	Poor
[30] (2020) India	+	+	?	?	+	+	?	Fair
[31] (2022) India	+	+	?	?	+	+	+	Fair
[32] (2019) Netherlands	+	+	−	?	+	+	+	Fair
[33] (2019) Netherlands	+	+	−	?	+	+	+	Fair
[34] (2018) Spain	−	?	?	+	−	+	−	Poor
[35] (2019) Mexico	−	−	−	?	+	+	No control group	Poor
[36] (2018) Italy	?	?	?	?	+	+	?	Poor
[37] (2022) USA	?	?	?	?	+	+	No control group	Poor
[38] (2020) UK	+	+	+	?	+	+	?	Fair
[39] (2021) Germany	−	?	?	?	+	+	No control group	Poor
[40] (2017) Lebanon	−	?	?	?	+	+	No control group	Poor

Note: (+) shows low risk of bias; (−) shows high risk of bias; (?) shows unclear risk of bias.

**Table 3:** Characteristics of study participants.

Studies’ General Information	Total Number of Participants/Numbers of Participants in Intervention Group/Control Group	Demographic Characteristics	Significant Baseline Differences between Groups	Duration of Diabetes Diagnosis	Setting(s)
[22] (2019) China	97 participants IG: 49 CG: 48	Average age: 63.71 years	No significant differences between groups	Diagnosis received within the prior 3 months	Outpatient ward
[23] (2018) China	118 participants IG: 63 CG: 55	Mean age: 54 ± 11.5	No significant differences between groups	Newly diagnosed T2DM	Outpatients and inpatients
[24] (2018) China	106 patients IG: 53 CG: 53	Mean age: 58.62 ± 15.74 years old	No significant difference between groups	1.24 years ± 0.35 years	Hospitalized patients
[25] (2019) China	358 participants IG: 179 CG: 179	Mean age of 50.1 ± 9.1 years	No significant difference between the two groups (*p* > 0.05)	Newly diagnosed T2DM	Outpatient ward
[26] (2022) China	128 participants IG: 64 CG: 64	Average age: 57.43 years, and 41.4% were female	No significant differences between groups	Newly diagnosed T2DM (Diagnosed in the preceding 3 to 9 months)	Primary healthcare services
[27] (2018) China	126 participants IG: 66 CG: 60	Mean age: 32.71 ± 5.69	No significant differences between groups	Newly diagnosed T2DM (Duration < 6 months)	Outpatients
[28] (2019) India	248 participants IG: 126 CG: 122	The mean age was 43 ± 8.7 years, and 32.3% were women.	No significant differences between groups	Newly diagnosed T2DM	Outpatient ward
[29] (2018) India	96 participants IG: 48 CG: 48	The age group was from 25 to 65 years.	Not clear	Newly diagnosed T2DM	Medical outpatient ward
[30] (2020) India	136 participants IG: 66 CG: 70	Mean ages (IG: 37.2 ± 4.09 and CG: 37.55 ± 4.29); 41.89% were female	Not clear	Newly diagnosed T2DM	Medical outpatient ward
[31] (2022) India	66 participants IG = 33 CG = 33	Mean age: 42.29 ± 9.5; 66.66% Male	No significant differences between groups	Newly diagnosed T2DM (Within 3 months of diagnosis)	Endocrinology outpatient ward
[32] (2019) Netherlands	108 participants IG: 56 CG: 52	Mean age: 62.3 ± 7.8	There were some differences between groups in the prevalence of diabetes-related complications.	Newly diagnosed T2DM (Diabetes duration from 3 months to 5 years)	Outpatient ward
[33] (2019) Netherlands	137 participants IG: 62 CG: 75	Mean age: 63.6 (10.2)	No significant differences between groups	Newly diagnosed T2DM (Diabetes duration 1–3 years)	Outpatients
[34](2018) Spain	271 participants IG: 134 CG: 137	IG 65.6 ± 10.6 CG 67.5 ± 13.5	Not clear	Newly diagnosed T2DM, 1 year after diagnosis	Primary health centers
[35] (2019) Mexico	288 patients who had followed the program No control group	The mean age was 51.1 ± 10.3 years, and 56.2% were women.	Not clear	≤5 years after diagnosis with T2DM	Outpatient ward
[36] (2018) Italy	95 participants IG: 47 CG: 48	Mean age: 58.43 ± 7.34	No significant differences between groups	Newly diagnosed T2DM (Diagnosed within the previous 12 months)	Outpatient ward
[37] (2022) USA	17 participants No control group	Mean age: 52 years	Not clear	Newly diagnosed T2DM (Diagnosed within the past 12 months)	Outpatients
[38] (2020) UK	18 participants IG: 9 CG: 9	Mean age: 56 (6.5); F/M: 50%/50%	No significant differences between groups	Newly diagnosed T2DM (Diagnosed within the past 4 years	Outpatients
[39] (2021) Germany	24 participants No control group	Mean age: 56 (6.5); F 58%	-	Newly diagnosed T2DM (Diabetes duration < 4 years)	Outpatients
[40] (2017) Lebanon	75 participants No control group	Mean age: 55 ± 10.7; 552% Female	-	Newly diagnosed T2DM	Diabetes outpatient clinics

**Table 4:** Characteristics of the educational interventions evaluated in the studies included in the review.

Studies’ General Information	Type of Study	Type of Intervention	Follow-up Period	Characteristics of Educational Interventions					Theoretical Basis	Assessed Outcomes
				Educational Sessions, Duration	Delivery	Provider	Covered Self-Care Topics	Supporting Strategy		
[22] (2019) China	Experimental design/RCTs	Health promotion–multi-intervention program Individual intervention	18 months	Different return visit times for each patient Mobile application	Face-to-face and online	Team	General information for diabetes Self-care	Mobile and tablets	AADE 7 Self-Care Behaviors program	Biomedical outcomes Psychosocial outcomes
[23] (2018) China	Experimental design	Education program–multi-intervention program Group education	6 months	2 lecture sessions and interactive sessions	Face-to-face	Nurses	General information for diabetes Self-care	Lecturing, audio-visual, and discussion approach	Problem-based learning	Biomedical outcomes Psychosocial outcomes
[24] (2018) China	Intervention study	Education program–multi-intervention program Group education “One-to-one” health education	3 months	Not clear	Face-to- face	Nurses	General information for diabetes Self-care	none	Orem’s self-care theory	Biomedical outcomes Psychosocial outcomes
[25] (2019) China	Non-randomized controlled study	Implementation of a model Group education	6 months	The first education was outpatient education. Follow-up telephone visits	Face-to-face and phone call visits	Team	General information for diabetes Self-care	Learning manual, video tutorials, phone calls, diabetes clubs, meetings	Omaha System-based integrated nursing management model	Biomedical outcomes Psychosocial outcomes
[26] (2022) China	RTCs	Multi-intervention program Group education	12 weeks	8 interactive educational sessions	Face-to-face	Nurses	General information for diabetes Self-care	Handbook and PowerPoint slides	Health belief model and Self-efficacy theory	Biomedical outcomes Psychosocial outcomes Behavioral outcomes
[27] (2018) China	Prospective cohort study	Education program–multi-intervention program Individual intervention Mobile application	24 weeks	Use of the medical app to assist in doctor–patient communication,	Online	Physician	General information for diabetes Self-care	Use of other functions of the app software	None	Biomedical outcomes
[28] (2019) India	RCTs	Education program–multi-intervention program Individual intervention	24 months	Advice from 2–3 educatory text messages per week	Text message Face-to-face	Physicians	General information for diabetes Self-care	None	None	Biomedical outcomes Psychosocial outcomes Behavioral outcomes
[29] (2018) India	RCTs	Education program–multi-intervention program Group intervention	4 months	1 session for 7–15 min	Face-to-face	Not clear	General information for diabetes Self-care	Video leaflets	None	Psychosocial outcomes
[30] (2020) India	RCTs	Structured exercise therapy Individual intervention	6 months	Aerobic exercise	Face-to-face	Physicians	Role of specific exercise	Booklets	None	Biomedical outcomes
[31] (2022) India	RCTs	Physical promotion Individual intervention	12 months	Contacted by phone at 3-month intervals	Calls and face-to-face	Not clear	Role of physical activity	No information	None	Biomedical outcomes Behavioral outcomes
[32] (2019) Netherlands	RCTs	Education program–multi-intervention program Individual and group intervention	30 months	two individual and five group sessions	Face-to-face	Nurses	General information for diabetes Self-care	Telephone consultation,	None	Biomedical outcomes Psychosocial outcomes Behavioral outcomes
[33] (2019) Netherlands	RCTs	Education program–multi-intervention program Group intervention	8 months	Three monthly 2-h interactive sessions and one booster session	Face-to-face	Nurses	Illness perceptions	Workbook for both patients and partners.	None	Psychosocial outcomes Behavioral outcomes
[34] (2018) Spain	Prospective study	PAET-Debut DM2 Standardized group education Group education	12 months	Three phases	Face-to-face	Team	General information for diabetes Self-care	Standardized materials	AISBE group for chronic diabetes disease	Biomedical outcomes Psychosocial outcomes Behavioral outcomes
[35] (2019) Mexico	Program evaluation study	Implementation of the CAIPadi model Individual intervention	24 months	Intervention visits and two follow-up visits (12 and 24 months)	Face-to-face	Team	General information for diabetes Self-care	Support in distance system webpage	CAIPaDi program	Biomedical outcomes Psychosocial outcomes Behavioral outcomes
[36] (2018) Italy	Intervention study	Psychological intervention Group intervention	12 months	90 min biweekly group sessions over 3 months	Face-to-face	Clinical psychologist	General information for diabetes Self-care	None	No	Biomedical outcomes
[37] (2022) USA	Intervention study	Education program–multi-intervention program Individual intervention	6-week intervention and 3-month follow-up	Four sessions	Face-to-face	Not clear	Role of foods and physical activity	The GEM pocket guide; Text messages	None	Biomedical outcomes Psychosocial outcomes Behavioral outcomes
[38] (2020) UK	RCTs	Education program–multi-intervention program Individual intervention	8 weeks	Participants received two text messages per week	Online	Not clear	Behavioral change	Use of a mobile application	Theory of planned behavior	Behavioral outcomes
[39] (2021) Germany	Intervention study	Food-based digital education Individual intervention Mobile application	12 weeks	During the weekly coaching calls	Online	Trained nutritionist	Structured behavioral change Role of a healthy diet	Recipe book	None	Biomedical outcomes
[40] (2017) Lebanon	Descriptive pre-/poststudy	Food education Individual intervention Mobile platform	12 months	5 visits	Face-to-face and online	Six Lebanese dietitians	General information for diabetes Self-care	No information	Academy of Nutrition and Dietetics EBNPGs	Biomedical outcome Behavioral outcomes

**Table 6:** The effects of educational interventions on anthropometric parameters.

Studies’ General Information	Pre-Intervention		Post-Intervention		Change		Absolute Effect
	IG	CG	IG	CG	IG	CG	
Effect of educational intervention on weight							
[22]	67.86 ± 16.84	66.67 ± 17.28	57.5 ± 15.33 *	65.65 ± 16.98	−10.36 **	−1.02	−9.34
[31]	71.47 ± 11.43	71.47 ± 11.43	69.27 ± 13.88	71.20 ± 12.76	−2.2	−0.27	−1.93
[32]	88.2 ± 16.2	87.7 ± 15.4	86.6 ± 16.1	86.7 ± 14.1	−1.6	−1	−0.6
[38]	89.60 ± 20.3	90.2 ± 19.9	90 ± 21.7	90.5 ± 19.2	+0.4	+0.3	+0.1
[39]	97.0 ± 13.9	-	87.7 ± 12.1 *	-	−9.3		−9.3
Mean ± SD					−4.6	−0.49	−2.94
Effect of educational intervention on BMI							
[22]	25.47 (3.31)	25.29 (3.25)	25.28 (2.93)	24.94 (2.89)	−0.19	−0.35	−0.16
[23]	25.70 ± 3.38	25.06 ± 3.38	25.16 ± 3.38	25.28 ± 3.47	−0.54	+0.02	−0.56
[27]	26.27 ± 4.64	25.52 ± 4.76	25.68 ± 4.21	25.48 ± 4.65	−0.59	−0.04	−0.55
[31]	27.73 ± 5.14	26.80 ± 2.76	26.97 ± 5.04	26.66 ± 3.18	−0.76	−0.14	−0.62
[32]	29.6 (4.9)	30.1 (4.5)	29.2 (4.8) *	29.6 (4.5)	−0.4	−0.5	−0.1
[34]	29.6 (27.2–34.2)	-	28.8 (25.9–32.6) *	-	−0.8 *		−0.8
[35]	29.19 ± 4.27	-	28.8 ± 4.2	-	−0.39		−0.39
[37]	36.5 (8.1)	-	34.4 (8.2)	-	−2.1		−2.1
[38]	31.1 ± 6.4	29.9 ± 4.7	31.2 ± 6.9	31.2 ± 4.6	+0.1	+1.3	−
[39]	32.6 ± 4.6	-	29.4 ± 3.9 *	-	−3.2		−3.2
[40]	31.7 ± 4.9	-	30.6 ± 4.9 *	-	−1.1 ± 2.7 *		−1.1
Mean ± SD					−0.90	0.29	−0.398

Notes: * Significant difference between pre- and post-intervention in the same group. ** Significantly different effect between IG and CG. Studies without a control group are not included in the calculation of the absolute effect on anthropometric parameters.

**Table 7:** The effects of educational interventions on blood pressures.

Blood Pressure/Studies’ General Information			[22]	[23]	[31]	[32]	[34]	[35]	[38]	[40]	Mean ± SD
SBP	Pre-intervention	IG	130.24 ± 18.92	130.00	128.09 ± 10.78	132 ± 13	130.64 ± 13.5	128.9 ± 16.4	136.3 ± 17.2	131.3 ± 20.4	
		CG	128.09 ± 17.36	120.00	129.48 ± 10.71	133 ± 14	-	-	134.0 ± 18.1		
	Post-intervention	IG	131.37 ± 19.12	130.00	119.15 ± 7.75	135 ± 17	129.4 ± 14.4	120.86 ± 11.83	138.2 ± 20.7	124.9 ± 9.9 *	
		CG	131.15 ± 18.24	130.00	119.88 ± 7.31	135 ± 15	-	-	135.6 ± 20.3		
	Change	IG	1.13	0	−8.94	3.0	1.24	−8.04	1.9	−6.4 *	−2.3 ± 6.5
		CG	3.06	10.00	−9.6	3			1.6		1.61 ± 8.10
	Absolute Effect			-	−0.66	0	-	−8.04	-	−6.4 *	−0.34 ± 7.9
DBP	Pre-intervention	IG	74.99 ± 13.12	80.00	79.33 ± 8.14	-	77.8 ± 9	78.4 ± 7.87	83.8 ± 9.5	81.3 ± 12.5	
		CG	76.22 ± 12.11	80.00	81.24 ± 8.03	-	-		83.3 ± 10.7		
	Post-intervention	IG	75.58 ± 11.04	80.00	78.15 ± 4.83	-	76.4 ± 8.4	74.06 ± 6.86	82.4 ± 9.8	78.1 ± 9.5	
		CG	78.61 ± 12.98	85.00	78.73 ± 5.78	-	-	-	84.3 ± 13.2		
	Change	IG	−0.59	0	1.18 ± 6.48	-	−1.4	4.34	−1.3	−3.1 *	−0.87 ± 4.5
		CG	2.39	0.5	2.51 ± 6.90	-			1.0		1.6 ± 5.1
	Absolute Effect		3.98	0.5	1.33	-	-	4.34	2.3	-3.1	0.36 ± 5.5

Notes: * Significant difference between pre- and post-intervention in the same group.

## Data Availability

Data sharing does not apply to this article as no datasets were generated or analysed during the current study.
